# Ensembles Are Required to Handle Aleatoric and Parametric Uncertainty in Molecular Dynamics Simulation

**DOI:** 10.1021/acs.jctc.1c00526

**Published:** 2021-07-19

**Authors:** Maxime Vassaux, Shunzhou Wan, Wouter Edeling, Peter V. Coveney

**Affiliations:** † Centre for Computational Science, Department of Chemistry, University College London, London WC1H 0AJ, United Kingdom; ‡ Centrum Wiskunde & Informatica, Scientific Computing Group, Amsterdam 1090 GB, The Netherlands; § Informatics Institute, University of Amsterdam, Amsterdam 1012 WX, The Netherlands

## Abstract

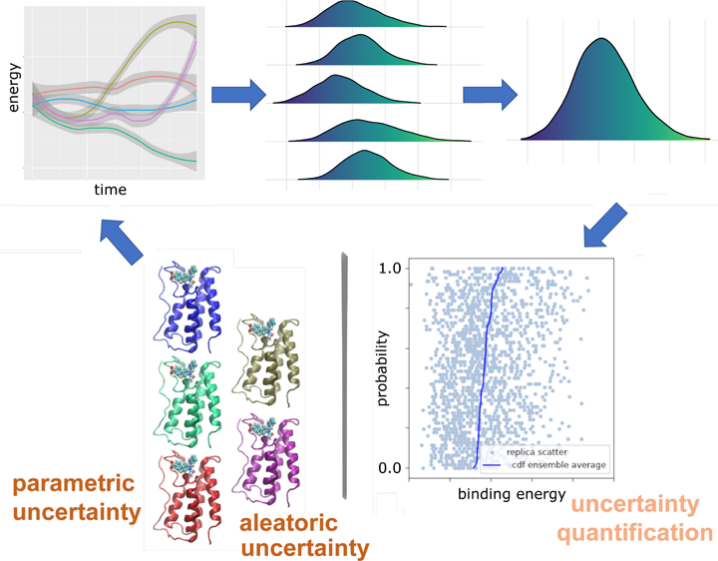

Classical molecular dynamics is a computer simulation technique that is in widespread use across many areas of science, from physics and chemistry to materials, biology, and medicine. The method continues to attract criticism due its oft-reported lack of reproducibility which is in part due to a failure to submit it to reliable uncertainty quantification (UQ). Here we show that the uncertainty arises from a combination of (i) the input parameters and (ii) the intrinsic stochasticity of the method controlled by the random seeds. To illustrate the situation, we make a systematic UQ analysis of a widely used molecular dynamics code (NAMD), applied to estimate binding free energy of a ligand-bound to a protein. In particular, we replace the usually fixed input parameters with random variables, systematically distributed about their mean values, and study the resulting distribution of the simulation output. We also perform a sensitivity analysis, which reveals that, out of a total of 175 parameters, just six dominate the variance in the code output. Furthermore, we show that binding energy calculations dampen the input uncertainty, in the sense that the variation around the mean output free energy is less than the variation around the mean of the assumed input distributions, if the output is ensemble-averaged over the random seeds. Without such ensemble averaging, the predicted free energy is five times more uncertain. The distribution of the predicted properties is thus strongly dependent upon the random seed. Owing to this substantial uncertainty, robust statistical measures of uncertainty in molecular dynamics simulation require the use of ensembles in all contexts.

## Introduction

The classical molecular dynamics computer simulation technique, which solves Newton’s equations of motion for assemblies of molecules, is a very widely used method across all areas of scientific research, from physics and chemistry to materials, biology, and medicine. Today it is commonplace to read reports of such simulations being performed on models containing many tens of thousands of atoms, and in the largest cases as many as some hundreds of millions of atoms as in the 2020 Gordon Bell award in the COVID-19 category for simulation of the Spike protein.^1^ What is clear, however, is that despite such studies abounding in the academic research literature, their impact in contexts where decision-making is required are few and far between. That is to say, the method is rarely used to make actionable decisions—ones which are taken as a matter of urgency based on the predictions of a computer simulation. While this is done routinely in many engineering contexts in which macroscopic simulations are performed, it remains uncommon at the molecular and lower length and time scales. In general, molecular dynamics is regularly used as a kind of post hoc rationalization method to explain experimental observations after they have occurred.

A well-known application of molecular dynamics involves the prediction of the binding affinity of a lead compound or drug candidate with a protein target, which is of central importance in drug discovery and personalized medicine. The binding affinity, also known as the free energy of binding, is the single most important initial indicator of drug potency, and the most challenging to predict.^2,3^ There are various approaches to estimate the magnitude of the binding free energy (a measure of how strong the interaction is between a ligand and its target protein), based on different theories and approximations.^4^ Molecular mechanics Poisson–Boltzmann surface area (MMPBSA) and molecular mechanics generalized Born surface area (MMGBSA) methods^5^ are among the most popular methods for free energy calculations, which are based on invoking a continuum approximation for the aqueous solvent to approximate electrostatic interactions following all-atom molecular dynamics simulations. There are other approaches with different approximations, domains of application, and computational requirements. The choice of which computational method to use is influenced by the desired accuracy, precision, time to solution, computational resources available, and so on. Even today, all these methods remain prone to sizable errors and are deemed unreliable for decision-making.

To make progress toward actionable molecular dynamics simulations, several things are required. The first is to ensure that the methods being used are reproducible, an essential requirement for any scientific method.^6−8^ Beyond that, the methods need to be validated against experiment, and verified in the sense that the codes used are indeed implementing the correct mathematics. Finally, codes should be subjected to an uncertainty quantification (UQ) study, in order to report the magnitude and distribution of the uncertainty which is inherently present.

There are two sources of uncertainty accruing in MD simulations, due to systematic and random sources.^7^ In order to get a full grip on uncertainty in MD simulations, one needs to be able to identify and quantify both. Epistemic uncertainty is introduced by inaccuracies inherent to the system investigated and within the measurement method performed. On the one hand, they come from the assumptions and approximations made when a theory is applied, a model is constructed, or a process is mimicked by the simulation of a real-world problem. In principle, a higher level of resolution should produce more accurate predictions than a lower level one, although in practice it is not always the case because of the quality of the theory employed.^9−11^ On the other hand, systematic errors can arise from the calibration of the MD engine. The thermodynamic conditions, such as constant volume or pressure in a closed system, must be accurately specified. Multiple factors need to be carefully considered in the preparation of the molecular models, such as choice of force field, protonation and tautomeric states, buffer conditions, use of physical restraints and constraints, thermostat, and barostat.

Epistemic uncertainty can be tied to imperfectly known input parameters, and/or approximate mathematical models. This uncertainty can in principle be reduced via improved mathematical models, or by calibrating the parameters to data. However, random variation, also called system noise, aleatoric or stochastic uncertainty, is caused by the intrinsically chaotic nature of classical molecular dynamics. While this uncertainty cannot be reduced, it can be quantified via ensemble methods. Given the extreme sensitivity of Newtonian dynamics to initial conditions, two independent MD simulations will sample the microscopic states with different probabilities no matter how close the initial conditions used.^12^ The impact of the chaotic nature of MD has not been widely recognized in the MD field. Leimkuhler and Matthews’ book (2015)^13^ is a notable exception, although it does not address the issue of uncertainty quantification.

The parameters used in MD simulations are usually calibrated to reproduce one or more available measurements from experiments, calculations from quantum mechanics, or both. In almost all cases, only a single value is used for the parameters, while the uncertainty in the parameters is simply ignored. For a realistic model of a biomolecular system, the number of parameters is very large. There are ∼16 000 energy terms in the system we are studying here, excluding the terms for all of the water molecules. These energy terms contain ∼40 000 parameters. Only limited studies have been performed to quantify uncertainties from force field parameters, using relatively simple models such as TIP4P water molecules^14^ and/or focusing on a small subset of parameters such as those for the Lennard–Jones potential^15^ or the atomic radius and charge parameters.^16^ While a quantification of the uncertainties from all the force field parameters is beyond the scope of this work, we note that the above studies show that the prediction uncertainty arising from parameters may be larger than statistical simulation uncertainty.

**Figure 1:**
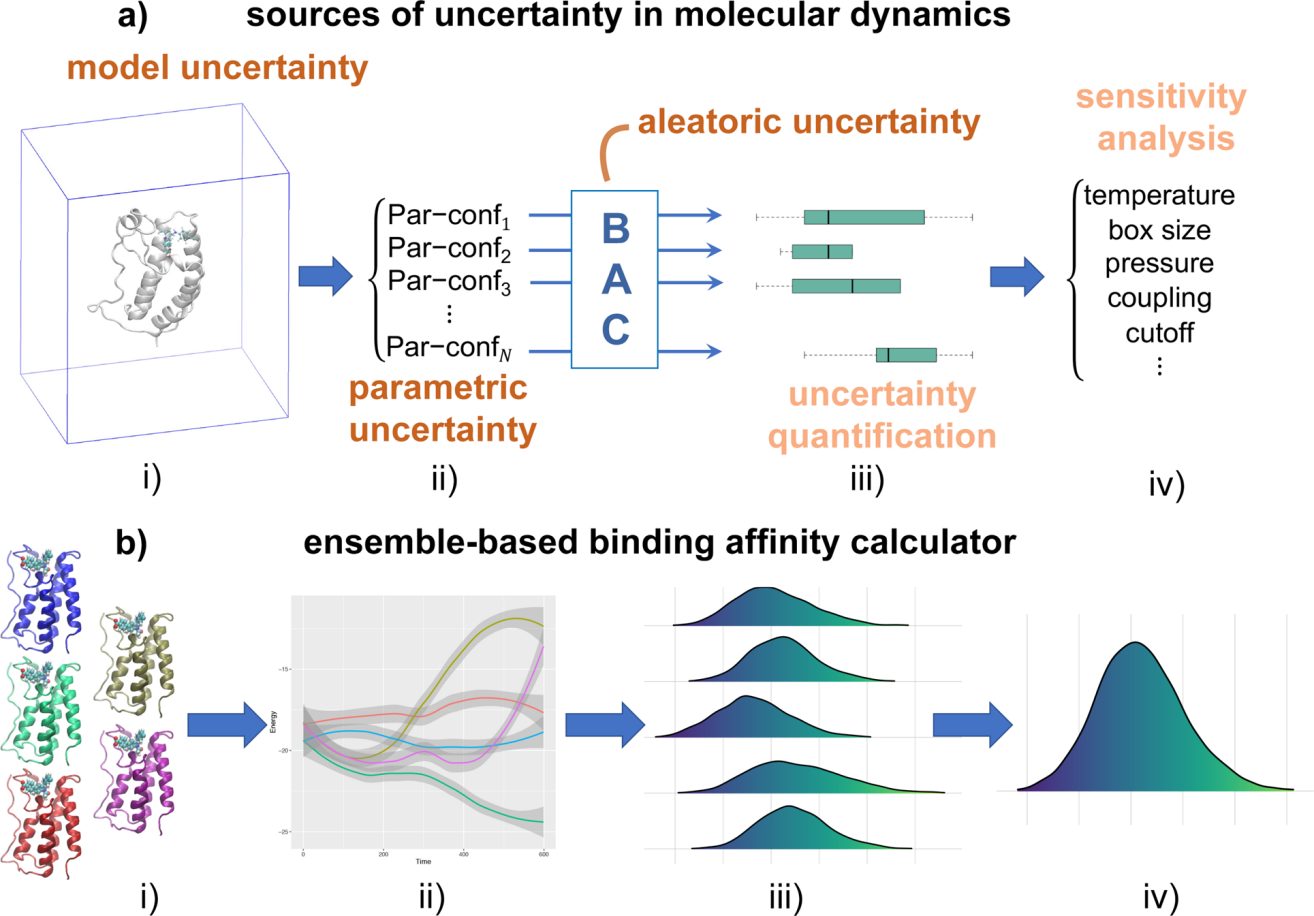
Sources of uncertainty and quality of predictions in molecular simulations for ensemble-based binding affinity calculations. (a) The types of uncertainties in the simulation (i) and the settings of parametric configurations (ii) are responsible for the uncertainty in predicted binding affinities (iii). Sensitivity analysis determines input parameters that most substantially impact predicted binding energy variability (iv). (b) The random errors are dealt with by ensemble approaches, in which multiple replicas (i) are simulated from initially close conformations. Neighboring trajectories in the “underlying” phase space diverge exponentially fast (ii), generating different distributions for a quantity of interest (iii). The number of replicas used to perform ensemble averaging (iv) varies, depending on the required accuracy and the power of the available computational resources.

In this paper, we perform such an uncertainty quantification study applied to a binding affinity calculation. Calculations are performed using Enhanced Sampling of Molecular dynamics with Approximation of Continuum Solvent (ESMACS)^17^ on a molecular complex of the bromodomain-containing protein 4 (BRD4-BD1) and the tetrahydroquinoline (I-BET726^18^) ligand (see Figure 1). In particular we perform a parametric UQ analysis, in which we replace deterministic scalar input parameters with random variables, and we also quantify the uncertainty arising from the seeds. Our overall goal is then to perform a forward propagation step, meaning we propagate the joint probability distribution of the inputs through NAMD via a suitable sampling method, in order to obtain the corresponding distribution of the simulation outcome. While NAMD has a large number of inputs (175) the majority of them are not relevant for forward UQ, as they do not directly influence the solution. Using expert knowledge, we selected a subset of 14 parameters which are known to have an impact on simulation behavior, to which we assigned uniform input distributions. It makes sense to reduce the number of input distributions a priori, since many forward UQ techniques (e.g., stochastic collocation (SC)^19^ or polynomial chaos expansions^20^) suffer from the curse of dimensionality. This essentially means that the required number of NAMD evaluations grows exponentially with the number of uncertain inputs, which leads to a computational bottleneck due to the compute-intensive nature of the code. This is further exacerbated due to the random seeds, which we also incorporate in our epistemic (parametric) uncertainty analysis. For each sample of the joint input distribution, we run 25 replica simulations in which we only vary the random seeds. One of our goals is to contrast the variation in the simulation outcome due to the parameters with the variation arising from the random seeds. We also examine the “robustness” of NAMD to epistemic uncertainty, by which we mean the extent to which the binding affinity calculation either damps or amplifies uncertainties from the input data to the output free energy predictions. Although we have *a priori* restricted the number of uncertain inputs, a 14-dimensional space is still too large to sample with standard SC or polynomial chaos expansions, while simple Monte Carlo is known to have a slow convergence rate. For this reason we employ a dimension-adaptive variant of the SC sampler.^21,22^ Briefly, this method banks on the existence of a low effective dimension, where only a subset of all parameters contribute significantly to the variance in the simulation output. The dimension-adaptive algorithm starts with a single sample, and iteratively refines the sampling plan along the directions which are found to be important, based on a suitable error metric. Details are given in the Theory and Methods section. Here we note that such methods have found application in a wide variety of domains, e.g. finance,^23^ natural convection,^24^ and epidemiology,^25^ to name just a few.

A final point of interest we wish to study here concerns the assumption of normality. From our investigations,^7^ we observe that the statistical properties one computes from molecular dynamics trajectories may be approximately described by a Gaussian random process. However, a normal distribution may not be automatically assumed. In fact, there are frequently significant deviations from such statistics in nonlinear dynamical systems of which molecular dynamics is an excellent example.^26,27^ The simulations should then proceed from a statistical-mechanical ensemble corresponding to the experimental conditions, and properties calculated from expected values may then be compared with their corresponding experimental counterparts. Following our recent findings, non-normality of binding free energies has been confirmed experimentally (Ian Wall and Alan Graves, private communication, 2020). Quantifying systematic errors requires first bringing the random components contributing to the errors under full control.

## Theory and Methods

We describe the ESMACS protocol, the dimension-adaptive sampling method, as well the methods to compute the Sobol index and uncertainty amplification factor. The last three methods are more extensively described in one of our previous studies of the CovidSim epidemiological code.^25^

### ESMACS Protocol and Ensemble Simulations

The protein target of our investigation is the bromodomain-containing protein 4,^17^ which is currently a major and rapidly evolving focus for the pharmaceutical industry. Preclinical and early stage clinical studies have shown that inhibitors targeting the protein exhibit promising efficacy in pathologies ranging from cancer to inflammation. BRD4 has recently become something of a benchmark system for free energy calculations, which we have investigated extensively using our binding affinity calculator for diverse compound data sets.^17,28^ Here we use one of the compounds studied previously,^17^ and investigate the sources of uncertainty along with the quality of binding free energy predictions.

The preparation and setup of the simulations are implemented using ESMACS. More details can be found in our previous publications.^17,29^ We use the same force field as described previously: the AMBER ff99SB-ILDN force field for the protein, TIP3P for water molecules, and the general AMBER force field (GAFF) for the ligand with partial charges calculated using restrained electrostatic potential (RESP) module in the AMBER package. The molecular system is solvated in orthorhombic water boxes. The minimal distance between the protein atoms and the box edges is set to be 14 Å as in our previous publications. It is treated here as one of the parameters included in the UQ study.

In the standard ESMACS protocol, an ensemble of 25 replicas is used for each of the parametric configurations. The starting phase spaces are close to each other for the replicas, differing only in their initial velocities which are generated independently from a Maxwell–Boltzmann distribution at 50K. Each molecular system is then virtually heated to a desired temperature, and subsequently maintained at this temperature and a defined pressure (with temperature and pressure coupling constants). After a total of 2 ns equilibration, a 4 ns production phase is initiated, of which the trajectory is analyzed to extract binding free energies. Full simulation details can be found in our previous publications.^17,29^

### Dimension-Adaptive Uncertainty Propagation

Our chosen method of propagating input uncertainty through NAMD is based on Stochastic Collocation (SC).^30^ Each input parameter  is assigned an independent probability density function *p*(ξ_*i*_), and the goal is to propagate these though NAMD to examine the corresponding distribution of the output. In particular, let  be the ensemble-averaged binding energy code output, computed at some parametric configuration  in the stochastic domain, as indexed by a multi-index (*j*_1_, ..., *j*_*d*_). Traditionally, the SC method involves an expansion over a tensor-product of such points, i.e.:
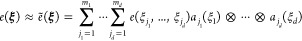
1

Here,  denotes the polynomial approximation of *e*, as each  is a 1D Lagrange interpolation polynomial given by the following:
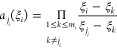
2

**Figure 2:**
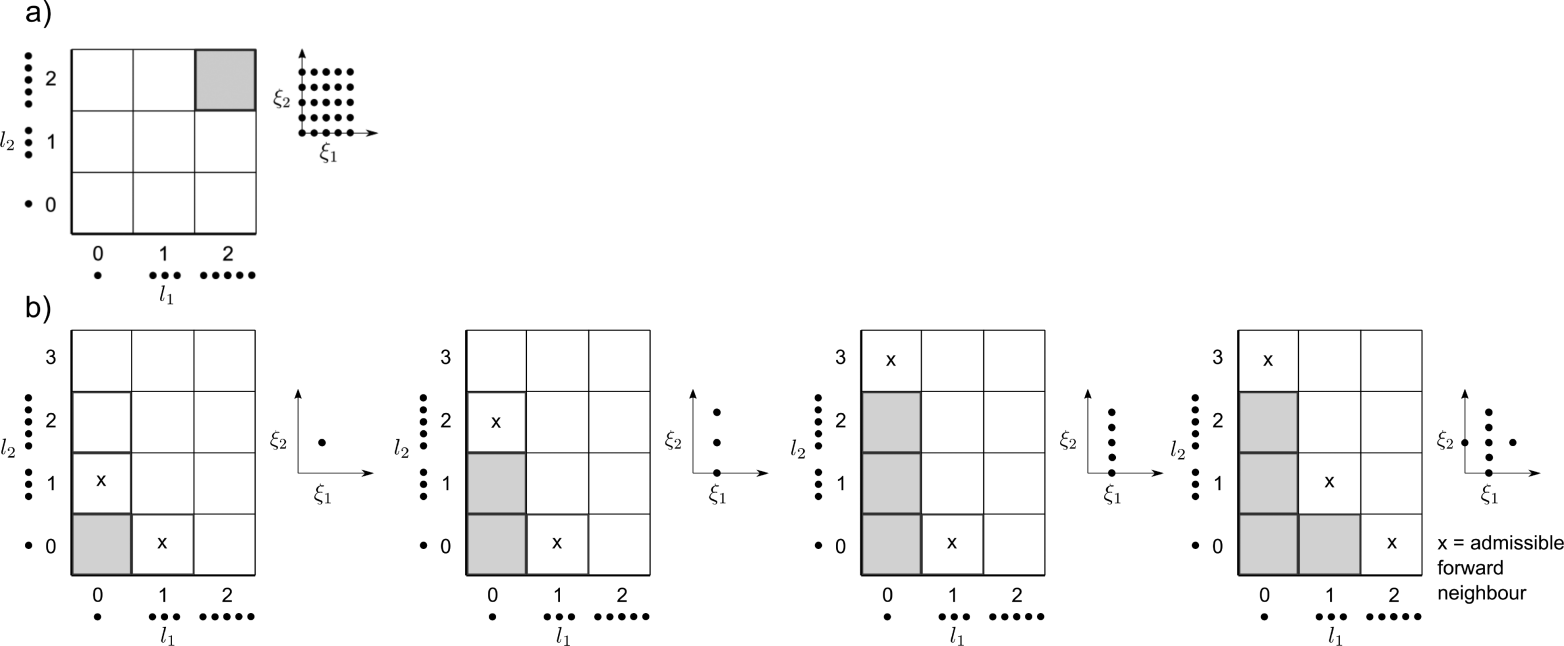
Two-dimensional examples of building sampling plans with one-dimensional quadrature rules of (different) orders. The horizontal axis displays the 1D quadrature points of order *l*_*i*_, and the corresponding sampling plan in (ξ_1_, ξ_2_) space is shown on the right. (a) A standard SC example, where the user specified a second-order rule for both inputs (**l** = (2, 2)), leading to a dense sampling plan of 25 points. (b) Possible iterations of a dimension-adaptive example. The first iteration contains the 0-th order rule for all inputs, i.e., Λ = {(0, 0)}. For this initial sampling plan there are two admissible candidate multi-indices, i.e., (1, 0) and (0, 1) (see × symbols). In this example, (0, 1) generated a larger error, and therefore gets accepted in Λ, leading to a more refined sampling plan in the ξ_2_ direction. This opens up new candidate directions, and the process repeats, leading to an anisotropic sampling plan. This plan is thus built from a linear combination of tensor products, using the quadrature orders in Λ.

A well-known property of the Lagrange polynomial associated with the *j*_*i*_-th collocation point (in a given dimension 1 ≤ *i* ≤ *d*), is that  at this point, and  at all other collocation points  (for *i* ≠ *k*). The 1D collocation points are generated from the points of a quadrature rule, used to approximate integrals weighted by the chosen input distribution *p*(ξ_*i*_). The order of this quadrature rule for the *i*-th input determines the number of points *m*_*i*_ along that dimension, and due to the tensor-product construction the total number of code evaluations for *d* inputs equals M = *m*_1_ · *m*_2_···*m*_*d*_, or M = *m*^*d*^ if all inputs receive the same quadrature order (see Figure 2a for an example). Note that, in the standard SC method, the order of each quadrature rule must be specified by the user. The exponential increase with the number of inputs *d* is known as the curse of dimensionality, and it limits practical applications of the standard SC method to less that about 10 uncertainty parameters. Since we have a 14 dimensional input space, we employed a dimension-adaptive version of the SC method, based on the original work of refs (19 and 21). This method does not remove the curse of dimensionality, although it does postpone its effect to higher dimensions. The general idea is to forego the standard single tensor product based on user-specified quadrature orders, and instead iteratively build the sampling plan using a linear combination of tensor products of different orders. Often, one starts from a single sample placed in the middle of the stochastic domain, which corresponds to assuming a 0-th order rule for all inputs. The sampling plan is then refined in an anisotropic fashion, sequentially increasing the order of (combinations of) inputs parameters which are deemed important by a suitable error metric. This method thus aims to find a lower effective dimension, which explains most of the variability of the output. While there is no guarantee of the existence of an effective dimension  with *K* < *d*, it is often observed in practice that only a small number of parameters are responsible for the majority of observed output variance, see e.g., ref (25). It should be noted that there are methods besides dimension-adaptive SC which also seek a lower effective dimension. Notable examples include High-Dimensional Model Representations,^31^ Active Subspaces,^32^ and more recent ideas involving machine learning.^33^

To adaptively refine the sampling plan, a “look-ahead step”^34^ is executed, where the computational model is evaluated at the new unique “candidate” locations which are admissible.^21^ The admissibility criteria is explained in detail by Gerstner et al. (2003);^21^ here we only provide a general outline. Let Λ be the set containing all quadrature-order multi indices **l** = (*l*_1_, ..., *l*_*d*_) which have been selected (the gray squares of Figure 2b), which, as stated, is initialized as Λ≔{(0, ..., 0)}. Now define the *forward neighbors* of any multi index **l** by the set {**l** + **e**_*i*_|1 ≤ *i* ≤ *d*}, where **e**_*i*_ is the elementary basis vector in the *i*-th direction, e.g., *e*_3_ = (0, 0, 1, ..., 0). The forward neighbors of the set Λ are then the forward neighbors for all *l* ∈Λ, which are not already in Λ. Similarly, the *backward neighbors* of **l** are given by {**l** – **e**_*i*_|*l*_*i*_ > 0, 1 ≤ *i* ≤ *d*}. An index set Λ is said to be *admissible* if all backward neighbors of Λ are in Λ. In short, the aforementioned candidate directions are generated by those forward neighbors **l** where Λ ∪{**l**} remains an admissible set, corresponding to the × symbols of Figure 2b. For each admissible forward neighbor **l**, a local error measure is computed. There are multiple possibilities for creating such a measure, either based on the interpolation error between subsequent levels of refinement,^22^ Sobol sensitivity indices^34^ or the observed error in quadrature metrics.^21^ For this study we adopt an error metric in the latter category where, similar to ref (35) we look for candidate directions defined by admissible multi indices, in which the change in variance is maximized. Hence, for every admissible multi-index **l** we compute a corresponding error measure ϵ_**l**_, defined as follows:
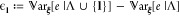
3

Here,  is the variance in the ensemble-averaged binding energy due to the uncertain inputs **ξ**, when evaluated using the points generated by the currently accepted multi indices in Λ. Likewise,  is the variance obtained if candidate multi-index **l** were to be accepted. Note that every index **l** = (*l*_1_, ..., *l*_*d*_) ∈Λ constitutes a separate tensor product of 1D quadrature rules with orders given by **l**. As noted, the SC expansion in the adaptive case is therefore constructed as a linear combination of tensor products over the accepted multi-indices in Λ, i.e.,
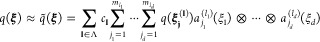
4where , and  is the number of points generated by a one-dimensional rule of order *l*_*i*_. The coefficients *c*_**l**_ are computed as
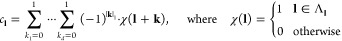
5see ref (19) for details. What remains is the specification of the type of 1D quadrature rule. In the case of (anisotropic) sparse grid methods as described here, it is common practice to select a *nested* rule, which has the property that a rule of a given order contains all points generated by that same rule at lower orders. When taking linear combinations of tensor products built from nested 1D rules of different order, as in (4), many points will overlap. This leads to a more efficient sampling plan in higher dimensions. For our calculations we employ the well-known Clenshaw-Curtis quadrature rule; see e.g., ref (22). Finally, we note that to generate the 1D rules, EasyVVUQ makes use of the Chaospy library.^36^

### Sobol Index Calculation

Briefly, the Sobol indices of  are global, variance-based measures of sensitivity of the ensemble-averaged binding energy *e* with respect to the inputs .^37,38^ It allows us to to identify important input parameters, and the indices have an intuitive interpretation. Let  be a so-called partial variance, where the multi-index **u** can be any subset of . Each partial variance represents the fraction of the total output variance that can be attributed to the input parameter combination indexed by **u**. When we normalize a partial variance with the total variance we obtain the corresponding Sobol index *S*_**u**_:
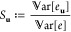
6where  is the is the total variance of *e*.^38^ Since all partial variances are positive, the sum of all possible *S*_**u**_ equals 1.

The number of all possible subsets **u** (the power set of ), rises exponentially with *d*. In practice, however, often only the first-order Sobol indices are computed, i.e., *S*_*i*_ with *i* ∈{1, ..., *d*}. These measure the variance fraction that can be attributed to each individual input, and more often than not, are already responsible for the majority of the output variance, such that the higher-order effects of varying multiple inputs simultaneously is relatively minor. This is also reflected in our results, see Section S4 of the Supporting Information (SI).

To compute the Sobol sensitivity indices, we employ the method described in ref (39). The general idea is to transform the adaptive SC expansion into a polynomial chaos expansion (PCE), which facilitates an easy computation of the Sobol indices. As this is already well documented, and not critical for our discussion, we refer to refs (25 and 39) for more details.

### Uncertainty Amplification Factor

In,^25^ we developed a “robustness score” for computational models, under uncertainty in the input parameters. Here, we modify it slightly to deal with negative in- and outputs. We base our robustness score on the coefficient of variation, a simple (dimensionless) measure for variability in some random variable *X*, defined as the standard deviation over the mean, i.e., CV(*X*) ≔ σ_*X*_/μ_*X*_. Any forward uncertainty propagation method approximates the first two moments of the output, and so the output CV is available. Assuming we can (analytically) compute the first two moments of each input ξ_*i*_ ∈**ξ**, *i* = 1, ..., *d*,  is also easily computed. As *d* > 1, we will compute the average variability at the input. Note that **ξ** may contain inputs defined on vastly different scales. Likewise, the order of magnitude between the input and output can also differ significantly. However, since the CV is a dimensionless quantity, this will not pose a problem. Here, we propose to use the ratio of the (absolute) CVs, denoted as CVR, as a relative measure of variability between the input and the output, which in the case of the scalar binding-energy becomes the following:
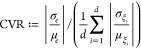
7

The absolute value is taken to avoid cancellation of variability. While technically not necessary in the case of NAMD, since all our inputs are positive and the output *e* is consistently negative, the current form of (eq 7) is more generally applicable in this fashion. Note that we do not include the random seed in the computation of the average input CV, since *e* here is the ensemble average over the replicas. In any case it would not make sense to compute the CV of the seeds, as the mean and variance of the random seeds are meaningless. Therefore, to still incorporate the effect of aleatoric uncertainty, we compute the output CV of each replica (CV(*e*_*i*_)) separately. and average these values afterward. In this case the CVR becomes
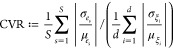
8where *S* is the number of random seeds considered, 25 in our case. The basic idea of eqs 7 and 8 is to say something about the robustness of the code to input uncertainty, given a user-specified input distribution. Note that Sobol indices are not suited for this goal. They attribute a fraction of the total output variance to subsets of parameters, and do not compare the variability observed at the output to the amount of variability assumed at the inputs. Thus, eqs 7 and 8 tell us to what extent the code amplifies the assumed input uncertainty, where we define amplification as having a CVR larger than 1. Relative damping occurs when CVR < 1, which is the case for our NAMD results.

## Results and Discussion

Binding affinity calculations performed by means of molecular dynamics simulations (using NAMD) depend on an extensive set of parameters. Exhaustively listing all possible parameters, we gathered 175 variables. However, not all these parameters should be included in the UQ procedure, and we use expert opinion to reduce this set.

### Dimension Reduction

A large number of parameters in the listing are configurational parameters; they control aspects such as I/O data flow but do not influence the behavior of the model simulation. Some parameters are also redundant between different equilibration and simulation phases of the affinity calculation. After eliminating these inputs, the listing was reduced down to 25 parameters. These remaining parameters can be classified into two groups:

- Group 1: “Physical parameters” which control the thermodynamics of the equilibration and binding processes; these essentially refer to the duration, the temperature and the pressure of the simulations (e.g., *setTemperature*, *BerendsenPressureTarget*, *time_sim1*).
- Group 2: “Solver parameters” which affect the algorithm used to compute the solution of the molecular dynamics equations; they modify the actual physics solved as well as the accuracy of the resolution (e.g., *initTemperature_eq1*, *time step*, *cutoff*).

From the physical parameters we selected a total of 4 parameters based on our experience with MD: temperature, pressure, equilibration duration, and sampling duration. Solver parameters were more numerous; there are 21 in total. However, 11 of these parameters are discrete variables which may not be suited for adaptive sampling methods, depending on the method used. Moreover, adding these parameters would drastically increase the cost of the UQ campaign. The 11 excluded parameters include: *reassignFreq* (frequency to reassign velocities of atoms to fit set temperature), *nonbondedFreq* (frequency to reevaluate nonbonded interactions), and *fullElectFrequency* (frequency to reevaluate electrostatics). Because of their influence on the solver behavior, we do not expect these parameters to have a strong impact on the binding affinity.

For the 14 remaining inputs, we choose uninformative uniform distributions to reflect our lack of knowledge in the most-likely values of these inputs, with bounds at ±15% from their nominal values. Only the temperature is also varied in a reduced range ([280 K, 320 K]) for physical reasons. These parameters and their uncertain ranges can be found in the SI (see Table S1).

### Uncertainty Quantification of Free Energy

The parametric configurations of the simulations, hence not the random seeds, are iteratively refined in directions where a variance-based error metric is largest (see the Theory and Methods section). Each iteration creates an ensemble of model evaluations, which we executed in parallel on the SuperMUC-NG supercomputer at the Leibniz-Rechenzentrum in Germany. We limited our study to the consumption of a budget of 2 000 000 CPUhs, which were allocated for this work. The computations were orchestrated using the VECMA Toolkit (VECMAtk),^40^ and specifically the EasyVVUQ library.^41,42^ Ensembles are chosen to contain a (large) number *N* of replicas such that adding one more replica does not change the statistical properties of the ensemble. The embarassingly parallel computations of ensembles is particularly suited for modern supercomputers. As NAMD is compute intensive, our strategy consisted of repeated refinement of the sampling plan until our computational budget was depleted. This occurred at 63 samples from the joint input probability distribution function in the reduced temperature range (123 samples in the full temperature range, see SI). For each sample, 25 replicas are simulated (using the same 25 seed values every time), each replica constituting an individual microstate. Their ensemble average corresponds to the thermodynamic macrostate. As a result, 1575 (3075 in the full temperature range) ESMACS workflow executions are completed for the purpose of this analysis. The use of an ensemble of replicas is standard in the field of UQ, in which a sufficiently large number of replicas are run concurrently from which reliable statistics can be extracted. Indeed, because molecular dynamics is intrinsically chaotic, the need to use ensemble methods is fundamental and holds regardless of the duration of the simulations performed. The number of replicas necessary in the ensemble varies from one system to the other and must be determined by direct investigation. Our previous studies show that, starting from reliable initial structures such as those obtained from high resolution crystallography experiments with extensive equilibration (each replica was separately equilibrated for 2 ns in the case of small proteins of approximately 150 amino acids), accurate and reproducible results can be achieved from ensemble simulations consisting of 25 replicas with 4 ns production runs.^4^

**Figure 3:**
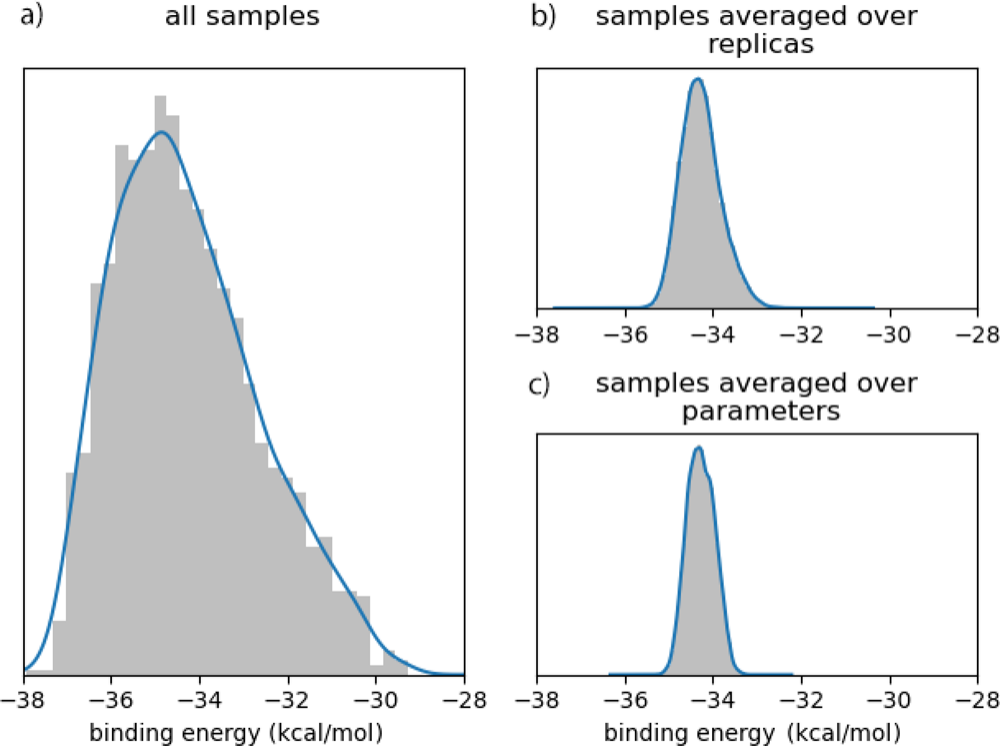
Non-normal distributions of computed binding free energies. (a) Distribution of the binding energies computed for each replica of each parametric configuration, resulting in 1575 samples in total. (b) Distribution of the binding energies averaged over the 63 parametric configurations for each of the 25 replicas. The distribution shows the influence of aleatoric uncertainty on the computed binding energies. (c) Distribution of the binding energies averaged over 25 replicas for each of the 63 parametric configurations. The distribution shows the influence of epistemic uncertainty on the computed binding energies. The continuous blue line corresponds to the kernel density estimator for each distribution.

The binding free energy is the quantity of interest of our UQ, the distribution of which follows a slightly asymmetric distribution peaking at −34.85 kcal/mol (based on the kernel density estimator of the distribution) with a longer tail for less negative binding energies (see Figure 3a). The standard deviation of the distribution is 1.63 kcal/mol. We also generate samples of averaged binding energies using bootstrapping, either averaged over replicas or parametric configurations, to analyze the respective contribution of epistemic and aleatoric uncertainty. However, the distribution of averaged binding energies over replicas (see Figure 3b)—that is for each parametric configuration the average of computed binding energies over 25 replicas—accounts solely for epistemic uncertainty. The non-normal distribution of ensemble-averaged energies reveals one peak around −34.36 kcal/mol with a thicker tail for less negative binding energy parametric configurations. The standard deviation is 0.45 kcal/mol. However, the distribution of averaged binding energies over parametric configurations (see Figure 3c)—that is the average of the computed binding energies over the 63 parametric configurations—accounts purely for aleatoric uncertainty. This distribution manifests a rather symmetric distribution centered around a peak at −34.35 kcal/mol as well. The distribution of parametric-averaged binding energies appears to be somewhat sharper than the ensemble-averaged ones, with a standard deviation of 0.31 kcal/mol. Nonetheless, the aleatoric uncertainty induces significant variations of the predicted binding energies. The standard deviation associated with the aleatoric uncertainty amounts to two-thirds of that associated with epistemic uncertainty. It should be noted however that the amount of epistemic uncertainty is directly linked to the assumed variance of the input distributions, such that the ratio of aleatoric to epistemic uncertainty changes with the input distribution of the parameters.

**Figure 4:**
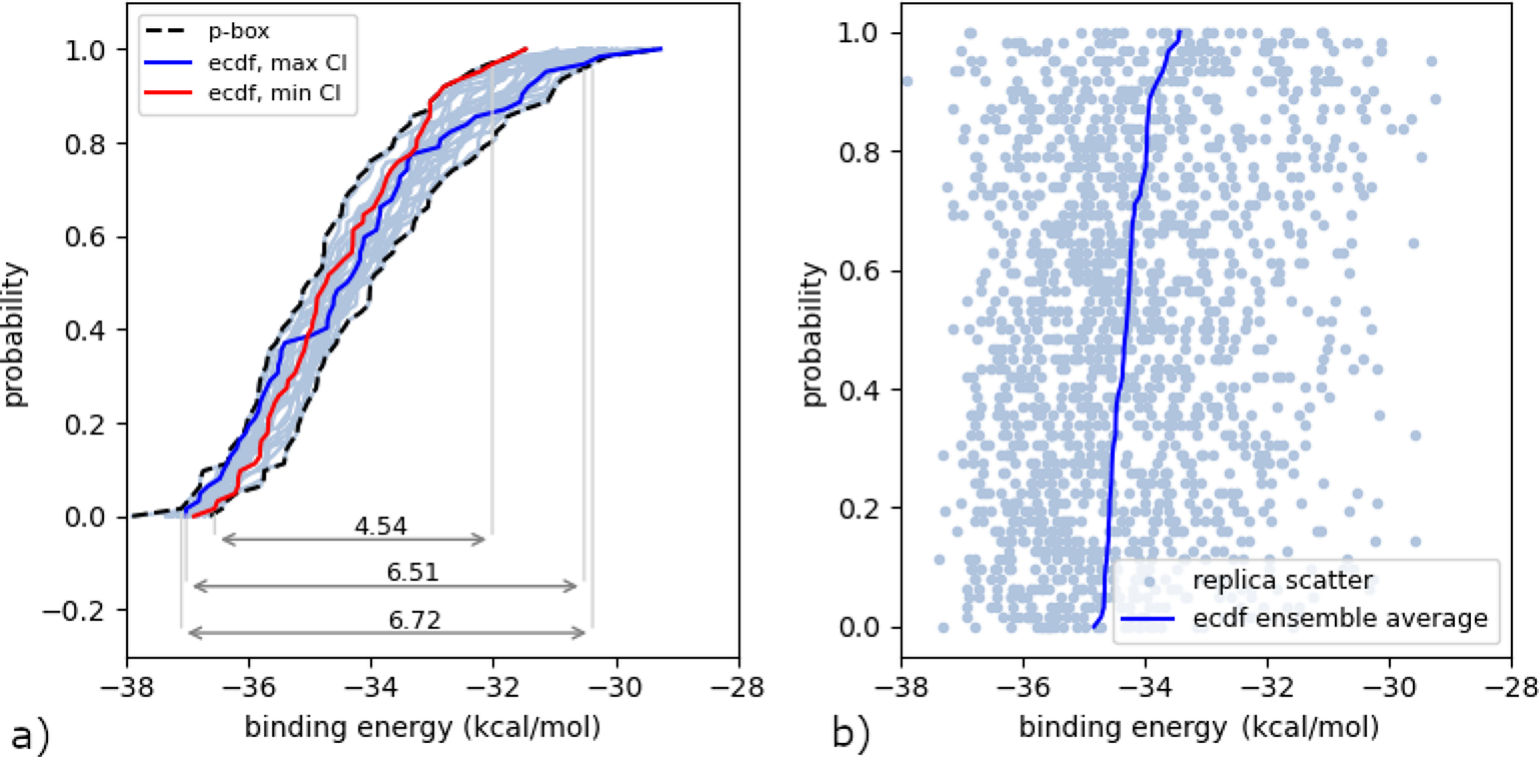
Effect of aleatoric uncertainty on the computed binding energy. (a) The probability box formed by the envelope of 25 ecdfs with fixed seed, with associated 95% confidence interval (CI, 6.72). In addition, the ecdfs with the largest/smallest (6.51/4.54) individual CIs are highlighted. (b) Cumulative distribution function of the ensemble-averaged binding energy of the 63 parametric configurations ensembles of 25 replicas (solid line); the individual dots on a given horizontal line show the individual binding energies of the replicas contributing to a given parametric configuration ensemble.

To provide further insights into the influence of aleatoric uncertainty, we investigate the distribution of binding energies within individual ensembles of replicas for a given parametric configuration. In particular, in Figure 4a we show a probability box (p-box) , where *e* denotes the binding energy. Let  be the cumulative distribution function (cdf) of the predicted binding energy when the random seed η is fixed to a given value η_*i*_, *i* = 1···25. The p-box is in this case then defined as the envelope formed by all 25 cdfs:
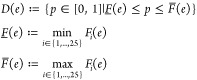
9

A p-box is commonly used to visualize possible outcomes due to a combination of epistemic and aleatoric uncertainty.^43^Figure 4a shows the p-box obtained from 25 empirical cdfs (ecdfs), each one estimated from 63 binding energy samples at a given random seed. The slant of each individual ecdf represents the epistemic uncertainty due to the different parameter values, whereas as the width of the p-box is governed by aleatoric uncertainty, caused by nonoverlapping ecdfs for different seeds. To extract 95% confidence intervals from the p-box we can simply form the interval , corresponding to  and , which gives us the displayed value of 6.72 kcal/mol. The width of the p-box already indicates the influence of aleatoric uncertainty. To further illustrate what could happen if we ignore the aleatoric uncertainty, we highlight two additional ecdfs in Figure 4a. These correspond to the maximum and minimum 95% confidence interval (CI) found in all 25 *individual* ecdfs. Thus, if we had fixed the seed to one of the 25 values we considered, and therefore executed the parametric UQ analysis without replicas, we could have obtained an estimated 95% CI of 4.54 kcal/mol, but a value of 6.51 kcal/mol would also have been possible, which is roughly a 30% difference. The p-box CI is more conservative as it combines both aleatoric and epistemic uncertainty.

To better visualize the spread of the predictions due to the seeds, consider Figure 4b. Each horizontal line of dots corresponds to one ensemble of replicas, ordered from bottom to top with increasing values of the mean binding energy of the ensemble. The solid line which links the mean binding energy of each of these ensembles corresponds to the ecdf of the ensemble-averaged energy of the 63 parametric configurations simulated. The aleatoric distribution of binding energies for a given parametric configuration is not constant. The shape of the distribution evolves with the mean binding energy of the parametric configuration.

**Figure 5:**
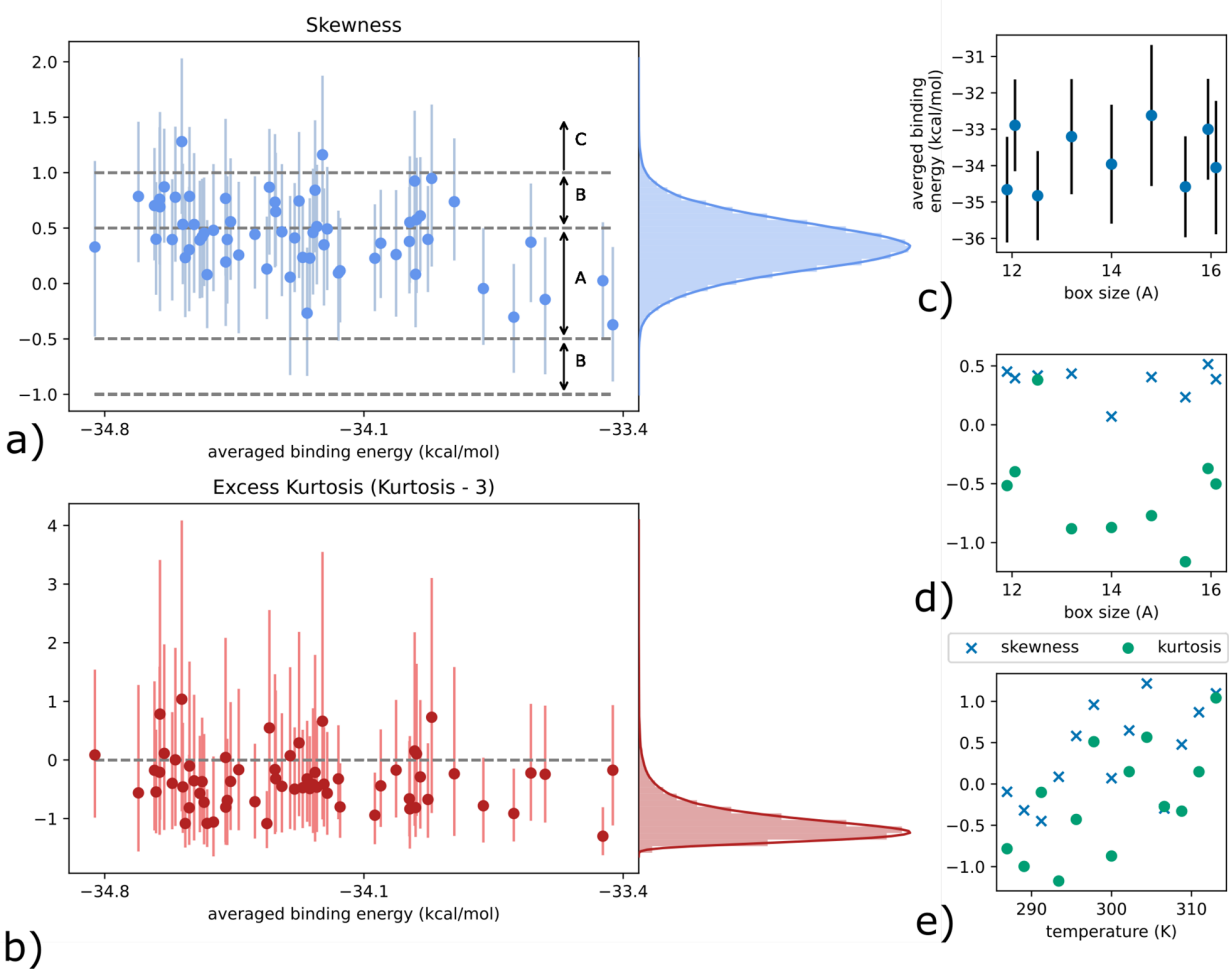
In-depth analysis of statistics: a loss of normality. (a) The skewness shape measure with 90% bootstrap confidence intervals, computed using the 25 replicas, for each of the 63 values of the ensemble-averaged binding energy. Region A corresponds to approximately symmetric distributions, region B to moderately skewed, and region C to significantly skewed distributions. The pdf of all samples is shown on the right. (b) An identical figure for the excess kurtosis shape measure. The horizontal line denotes the value of a standard normal distribution. (c) Mean binding energy for all parameters set to default except the box size (standard deviation as error bars). Skewness and kurtosis shape measures of the binding energy distributions (25 samples): (d) for all parameters set to default except the box size; (e) for all parameters set to default except the temperature.

This can be better shown via a more quantitative insight, provided by the analysis of the shape measures skewness and kurtosis, related to the third and fourth statistical moments, respectively. Skewness characterizes the symmetry of a distribution where, in the case of unimodal distributions, positive values indicate a distribution where the right tail is longer than the left. Kurtosis is related to the tails, where higher values indicate the presence of outliers in the distribution. Often, the so-called “excess kurtosis” is reported rather than the kurtosis itself, which is defined as kurtosis-3. Here, 3 is the value of kurtosis for a standard Gaussian distribution, such that the excess kurtosis measures a deviation with respect to this distribution. Our results are reported in Figure 5, where we display the skewness and excess kurtosis, with bootstrap confidence intervals, as a function of the value of the binding energy averaged over the replicas. For the skewness we make use of a common rule thumb^44^ to help with the interpretation of the numbers. Skewness values with an absolute magnitude smaller than 0.5 are said to be approximately symmetric, denoted by region A in Figure 5. Moderately skewed distributions correspond to absolute values in [0.5, 1.0] (region B), whereas absolute values which are >1 are said to indicate highly skewed distributions (region C). Despite large bootstrap confidence intervals, we can still observe a consistent trend, of (mostly) moderately (positively) skewed distributions for low averaged binding energy, that moves toward approximately symmetric distributions for higher averaged binding energies. In addition, we display the probability density function (pdf) of all bootstrap samples on the right of the figure. The average kurtosis value of this distribution is roughly 0.44, still within the approximately symmetric region. However, it is also clear that there is a significant nonzero probability of observing moderately (positively) skewed distributions. The excess kurtosis is consistently negative, meaning that compared to a normal distribution, the tails are shorter and thinner. Overall, these results imply the presence of non-normal distributions. Finally, we note that skewness and kurtosis appear uncorrelated with the box size (see Figure 5d), while they are linearly correlated with the temperature (see Figure 5e).

Our study shows that binding free energy is very sensitive to the temperature. This is not surprising as free energy is a temperature-dependent quantity according to the van’t Hoff equation. Reducing the size of molecular dynamics simulation cells is one of the most frequently used devices to reduce the expense of MD calculations. The effect of box size on the predicted thermodynamic and kinetic properties is currently the subject of an ongoing debate. In a recent study, a systematic change was reported for various predicted thermodynamic properties (averaged over 10 replicas) when the MD simulation box size was increased.^45,46^ Another study, however, found that the reported box size dependence was not reproducible when twice as many ensembles were used.^47,48^ Although box size is the second most sensitive parameter that our study reveals (see Figure S2), the calculated binding free energies do not change significantly (within error) when the box size varies (see Figure 5c). The SI contains more details on the influence of the other parameters when the contributions to uncertainty arising from the temperature parameter are removed (see Figure S3).

Finally, we compute the output variation relative to the mean, compared to the relative variation assumed at the input (see Table 1). This can be seen as a measure of the amount that the binding affinity calculation either amplifies or damps the assumed uncertainty from the input to the output. We base this on a measure which involves the ratio of the binding-energy coefficient of variation (CV(*e*)), with respect to the average input coefficient of variation ; see the Theory and Methods section. Briefly, a coefficient of variation (CV) is a dimensionless measure of variability, defined as the standard deviation over the mean. We can compute this for the binding energy *e*, and each of the *d* = 14 input parameters ξ_*i*_, taking the absolute value to avoid cancellation of variability. When  we say that the code amplifies input uncertainty, as the relative output variability exceeds that of the input. Conversely, damping occurs when CVR < 1.

**Table 1:** Coefficients of Variation^a^

ensemble averaging	CV	CV (*e*)	CVR
yes	0.087	0.0094	0.11
no	0.087	0.047	0.54

a The mean coefficient of variation (CV) for the input and the output and their ratio (CVR), with and without presence of ensemble averaging. In the model analysis, a CV of roughly 8% is introduced via inputs. The corresponding output variability is reduced down to 1% by the model when computing ensemble-averaged binding energies (over 25 replicas). When considering individual simulations, variability in the binding affinity is only reduced to 5%.

In our UQ campaign, the mean coefficient of input variation is about 8.5%. When considering ensemble-averaged binding energy estimations (over 25 replicas), the mean coefficient of variation of the binding affinity is less than 1%, leading to a CVR of 0.11. Such significant damping of uncertainty occurs when using the ensemble average binding energy as our quantity of interest. We can also consider the CVR we would obtain were we not to use ensemble averaging, by computing the mean of the individual binding-energy CVs over the 25 replicas, i.e., by using . As expected, the observed variability at the output is larger in this case, with a CV(*e*) of approximately 5%, leading to a CVR of 0.54. While we still consider this as a damping of uncertainty, it is roughly five times larger compared to the case where the binding energy is averaged over the 25 replicas. The use of ensembles of simulations therefore drastically reduces aleatoric uncertainty within binding affinity calculations, enabling a 5-fold decrease in the overall uncertainty within the model simulation in this case.

We conclude that the current practice of running one or only a small number of replicas of a molecular dynamics simulation is far from sufficient to control uncertainty, as already indicated in our previous studies.^4,49^ It does not enable one to control the error in the quantities of interest, as is achieved in a statistically robust manner by ensembles. We have previously drawn similar conclusions about the role of stochasticity in alchemical free energy methods including thermodynamic integration and free energy perturbation.^50^ Our findings apply to classical molecular dynamics simulation in general, including to all forms of free energy estimation made using it.^7^ The distributions of properties predicted using classical molecular dynamics cannot be assumed to be Gaussian but need to be assessed in each case, particularly when long-range interactions are involved.^4,7^ In general, means and standard deviations reported from a small number of repeated simulations will not be reliable. In conclusion, if we wish to produce actionable results from molecular dynamics simulations, whatever the predicted quantity of interest, we must invoke ensembles for which the use of modern supercomputers is essential.

## Supporting Information Available

Data used to generate the figures in the results section (ZIP)
Data used to generate the figures in the results section (ZIP)
Additional results which provide further information on aspects of the uncertainty in the binding affinity calculation workflow based on NAMD and on the parameter refinement performed; code used to generate our results available at a GitHub branch (https://github.com/UCL-CCS/UQ_NAMD/releases/tag/0.0.1); and scripts to perform the UQ campaign pushed to GitHub (PDF)

## References

[ref1] Casalino L.; Gaieb Z.; Goldsmith J. A.; Hjorth C. K.; Dommer A. C.; Harbison A. M.; Fogarty C. A.; Barros E. P.; Taylor B. C.; McLellan J. S.; Fadda E.; Amaro R. E. Beyond Shielding: The Roles of Glycans in the SARS-CoV-2 Spike Protein. ACS Cent. Sci. 2020, 6, 1722–1734. 10.1021/acscentsci.0c01056. 33140034 PMC7523240

[ref2] Dakka J.; Turilli M.; Wright D. W.; Zasada S. J.; Balasubramanian V.; Wan S.; Coveney P. V.; Jha S. High-throughput binding affinity calculations at extreme scales. BMC Bioinf. 2018, 19, 482 10.1186/s12859-018-2506-6. PMC6302294 30577753

[ref3] Wright D. W.; Coveney P. V. Resolution of Discordant HIV-1 Protease Resistance Rankings Using Molecular Dynamics Simulations. J. Chem. Inf. Model. 2011, 51, 2636–2649. 10.1021/ci200308r. 21902276

[ref4] Wan S.; Bhati A. P.; Zasada S. J.; Coveney P. V. Rapid, accurate, precise and reproducible ligand–protein binding free energy prediction. Interface Focus 2020, 10, 20200007 10.1098/rsfs.2020.0007. 33178418 PMC7653346

[ref5] Genheden S.; Ryde U. Comparison of the Efficiency of the LIE and MM/GBSA Methods to Calculate Ligand-Binding Energies. J. Chem. Theory Comput. 2011, 7, 3768–3778. 10.1021/ct200163c. 26598269

[ref6] Coveney P. V. Computational Biomedicine. Part 1: Molecular Medicine. Interface Focus 2020, 10, 20200047 10.1098/rsfs.2020.0047.

[ref7] Wan S.; Sinclair R. C.; Coveney P. V. Uncertainty quantification in classical molecular dynamics. Philos. Trans. R. Soc., A 2021, 379, 20200082 10.1098/rsta.2020.0082. PMC8059622 33775140

[ref8] Coveney P. V.; Groen D.; Hoekstra A. G. Reliability and Reproducibility in Computational Science: Implementing Validation, Verification and Uncertainty Quantification in Silico. Philos. Trans. R. Soc., A 2021, 379, 20200409 10.1098/rsta.2020.0409. 33775138

[ref9] Mobley D. L.; Wymer K. L.; Lim N. M.; Guthrie J. P. Blind prediction of solvation free energies from the SAMPL4 5 challenge. J. Comput.-Aided Mol. Des. 2014, 28, 135–150. 10.1007/s10822-014-9718-2. 24615156 PMC4006301

[ref10] Bannan C. C.; Burley K. H.; Chiu M.; Shirts M. R.; Gilson M. K.; Mobley D. L. Blind prediction of cyclohexane–water distribution coefficients from the SAMPL5 challenge. J. Comput.-Aided Mol. Des. 2016, 30, 927–944. 10.1007/s10822-016-9954-8. 27677750 PMC5209301

[ref11] Rizzi A.; Murkli S.; McNeill J. N.; Yao W.; Sullivan M.; Gilson M. K.; Chiu M. W.; Isaacs L.; Gibb B. C.; Mobley D. L.; Chodera J. D. Overview of the SAMPL6 host–guest binding affinity prediction challenge. J. Comput.-Aided Mol. Des. 2018, 32, 937–963. 10.1007/s10822-018-0170-6. 30415285 PMC6301044

[ref12] Coveney P. V.; Wan S. On the calculation of equilibrium thermodynamic properties from molecular dynamics. Phys. Chem. Chem. Phys. 2016, 18, 30236–30240. 10.1039/C6CP02349E. 27165501

[ref13] Leimkuhler B.; Matthews C. Molecular Dynamics: With Deterministic and Stochastic Numerical Methods; Springer International Publishing: Berlin, Germany, 2015.

[ref14] Rizzi F.; Najm H. N.; Debusschere B. J.; Sargsyan K.; Salloum M.; Adalsteinsson H.; Knio O. M. Uncertainty Quantification in MD Simulations. Part II: Bayesian Inference of Force-Field Parameters. Multiscale Model. Simul. 2012, 10, 1460–1492. 10.1137/110853170.

[ref15] Angelikopoulos P.; Papadimitriou C.; Koumoutsakos P. Bayesian uncertainty quantification and propagation in molecular dynamics simulations: A high performance computing framework. J. Chem. Phys. 2012, 137, 144103 10.1063/1.4757266. 23061835

[ref16] Yang X.; Lei H.; Gao P.; Thomas D. G.; Mobley D. L.; Baker N. A. Atomic Radius and Charge Parameter Uncertainty in Biomolecular Solvation Energy Calculations. J. Chem. Theory Comput. 2018, 14, 759–767. 10.1021/acs.jctc.7b00905. 29293342 PMC6906122

[ref17] Wan S.; Bhati A. P.; Zasada S. J.; Wall I.; Green D.; Bamborough P.; Coveney P. V. Rapid and Reliable Binding Affinity Prediction of Bromodomain Inhibitors: A Computational Study. J. Chem. Theory Comput. 2017, 13, 784–795. 10.1021/acs.jctc.6b00794. 28005370 PMC5312866

[ref18] Gosmini R.; Nguyen V. L.; Toum J.; Simon C.; Brusq J.-M. G.; Krysa G.; Mirguet O.; Riou-Eymard A. M.; Boursier E. V.; Trottet L.; Bamborough P.; Clark H.; Chung C.-w.; Cutler L.; Demont E. H.; Kaur R.; Lewis A. J.; Schilling M. B.; Soden P. E.; Taylor S.; Walker A. L.; Walker M. D.; Prinjha R. K.; Nicodème E. The Discovery of I-BET726 (GSK1324726A), A Potent Tetrahydroquinoline ApoA1 Up-Regulator and Selective BET Bromodomain Inhibitor. J. Med. Chem. 2014, 57, 8111–8131. 10.1021/jm5010539. 25249180

[ref19] Gerstner T.; Griebel M. Numerical integration using sparse grids. Numerical Algorithms 1998, 18, 209 10.1023/A:1019129717644.

[ref20] Xiu D.; Karniadakis G. E. The Wiener–Askey Polynomial Chaos for Stochastic Differential Equations. SIAM J. Sci. Comput. 2002, 24, 619–644. 10.1137/S1064827501387826.

[ref21] Gerstner T.; Griebel M. Dimension–Adaptive Tensor–Product Quadrature. Computing 2003, 71, 65–87. 10.1007/s00607-003-0015-5.

[ref22] Loukrezis D.; Römer U.; De Gersem H. Assessing the Performance of Leja and Clenshaw-Curtis Collocation for Computational Electromagnetics with Random Input Data. International Journal for Uncertainty Quantification 2019, 9, 33–57. 10.1615/Int.J.UncertaintyQuantification.2018025234.

[ref23] Judd K. L.; Maliar L.; Maliar S.; Valero R. Smolyak method for solving dynamic economic models: Lagrange interpolation, anisotropic grid and adaptive domain. J. Econ. Dyn. Control 2014, 44, 92–123. 10.1016/j.jedc.2014.03.003.

[ref24] Ganapathysubramanian B.; Zabaras N. Sparse grid collocation schemes for stochastic natural convection problems. J. Comput. Phys. 2007, 225, 652–685. 10.1016/j.jcp.2006.12.014.

[ref25] Edeling W.; Arabnejad H.; Sinclair R.; Suleimenova D.; Gopalakrishnan K.; Bosak B.; Groen D.; Mahmood I.; Crommelin D.; Coveney P. V. The impact of uncertainty on predictions of the CovidSim epidemiological code. Nature Comput. Sci. 2021, 1, 128–135. 10.1038/s43588-021-00028-9. 38217226

[ref26] Coveney P. V.; Dougherty E. R.; Highfield R. R. Big data need big theory too. Philos. Trans. R. Soc., A 2016, 374, 20160153 10.1098/rsta.2016.0153. PMC5052735 27698035

[ref27] Succi S.; Coveney P. V. Big data: the end of the scientific method?. Philos. Trans. R. Soc., A 2019, 377, 20180145 10.1098/rsta.2018.0145. PMC6388004 30967041

[ref28] Aldeghi M.; Heifetz A.; Bodkin M. J.; Knapp S.; Biggin P. C. Predictions of Ligand Selectivity from Absolute Binding Free Energy Calculations. J. Am. Chem. Soc. 2017, 139, 946–957. 10.1021/jacs.6b11467. 28009512 PMC5253712

[ref29] Wright D. W.; Wan S.; Meyer C.; van Vlijmen H.; Tresadern G.; Coveney P. V. Application of ESMACS binding free energy protocols to diverse datasets: Bromodomain-containing protein 4. Sci. Rep. 2019, 9, 6017. 30979914 10.1038/s41598-019-41758-1 PMC6461631

[ref30] Eldred M.; Burkardt J. Comparison of non-intrusive polynomial chaos and stochastic collocation methods for uncertainty quantification. 47th AIAA Aerospace Sciences Meeting Including the New Horizons Forum and Aerospace Exposition. 2009; p 976.

[ref31] Rabitz H.; Aliş O. General foundations of high-dimensional model representations. J. Math. Chem. 1999, 25, 197–233. 10.1023/A:1019188517934.

[ref32] Constantine P. Active Subspaces: Emerging Ideas for Dimension Reduction in Parameter Studies; SIAM: Philadelphia, PA, 2015.

[ref33] Tripathy R.; Bilionis I. Deep UQ: Learning deep neural network surrogate models for high dimensional uncertainty quantification. J. Comput. Phys. 2018, 375, 565–588. 10.1016/j.jcp.2018.08.036.

[ref34] Dwight R. P.; Desmedt S. G. L.; Omrani P. S. Sobol Indices for Dimension Adaptivity in Sparse Grids. Simulation-Driven Modeling and Optimization. Cham 2016, 153, 371–395. 10.1007/978-3-319-27517-8_15.

[ref35] Narayan A.; Jakeman J. D. Adaptive Leja Sparse Grid Constructions for Stochastic Collocation and High-Dimensional Approximation. SIAM J. Sci. Comput. 2014, 36, A2952 10.1137/140966368.

[ref36] Feinberg J.; Langtangen H. P. Chaospy: An open source tool for designing methods of uncertainty quantification. Journal of Computational Science 2015, 11, 46–57. 10.1016/j.jocs.2015.08.008.

[ref37] Sobol I. M. On sensitivity estimation for nonlinear mathematical models. Mat. Model. 1990, 2 (1), 112–118.

[ref38] Sobol I. M. Global sensitivity indices for nonlinear mathematical models and their Monte Carlo estimates. Mathematics and Computers in Simulation 2001, 55 (1), 271–280. 10.1016/S0378-4754(00)00270-6.

[ref39] Jakeman J. D.; Eldred M. S.; Geraci G.; Gorodetsky A. Adaptive multi-index collocation for uncertainty quantification and sensitivity analysis. Int. J. Numer. Methods Eng. 2020, 121, 1314–1343. 10.1002/nme.6268.

[ref40] Groen D.; Arabnejad H.; Jancauskas V.; Edeling W. N.; Jansson F.; Richardson R. A.; Lakhlili J.; Veen L.; Bosak B.; Kopta P.; Wright D. W.; Monnier N.; Karlshoefer P.; Suleimenova D.; Sinclair R.; Vassaux M.; Nikishova A.; Bieniek M.; Luk O. O.; Kulczewski M.; Raffin E.; Crommelin D.; Hoenen O.; Coster D. P.; Piontek T.; Coveney P. V. VECMAtk: A Scalable Verification, Validation and Uncertainty Quantification Toolkit for Scientific Simulations. Philos. Trans. R. Soc., A 2021, 379, 20200221 10.1098/rsta.2020.0221. PMC8059654 33775151

[ref41] Richardson R. A.; Wright D. W.; Edeling W.; Jancauskas V.; Lakhlili J.; Coveney P. V. EasyVVUQ: A Library for Verification, Validation and Uncertainty Quantification in High Performance Computing. Journal of Open Research Software 2020, 8, 11 10.5334/jors.303.

[ref42] Wright D. W.; Richardson R. A.; Edeling W.; Lakhlili J.; Sinclair R. C.; Jancauskas V.; Suleimenova D.; Bosak B.; Kulczewski M.; Piontek T.; Kopta P.; Chirca I.; Arabnejad H.; Luk O. O.; Hoenen O.; Weglarz J.; Crommelin D.; Groen D.; Coveney P. V. Building Confidence in Simulation: Applications of EasyVVUQ. Advanced Theory and Simulations 2020, 3, 1900246 10.1002/adts.201900246.

[ref43] Oberkampf W.; Roy C. Verification and Validation in Scientific Computing; Cambridge University Press: Cambridge, U.K., 2010.

[ref44] Bulmer M. Principles of Statistics; Dover Publications: NY, 1979.

[ref45] El Hage K.; Hédin F.; Gupta P. K.; Meuwly M.; Karplus M. Valid molecular dynamics simulations of human hemoglobin require a surprisingly large box size. eLife 2018, 7, e35560 10.7554/eLife.35560. 29998846 PMC6042964

[ref46] El Hage K.; Hédin F.; Gupta P. K.; Meuwly M.; Karplus M. Response to comment on ‘Valid molecular dynamics simulations of human hemoglobin require a surprisingly large box size’. eLife 2019, 8, e45318 10.7554/eLife.45318 31219783 PMC6586459

[ref47] Gapsys V.; de Groot B. L. Comment on ‘Valid molecular dynamics simulations of human hemoglobin require a surprisingly large box size’. eLife 2019, 8, e44718 10.7554/eLife.44718. 31219782 PMC6586461

[ref48] Gapsys V.; de Groot B. L. On the importance of statistics in molecular simulations for thermodynamics, kinetics and simulation box size. eLife 2020, 9, e57589, Publisher: eLife Sciences Publications, Ltd. 10.7554/eLife.57589 32812868 PMC7481008

[ref49] Knapp B.; Ospina L.; Deane C. M. Avoiding False Positive Conclusions in Molecular Simulation: The Importance of Replicas. J. Chem. Theory Comput. 2018, 14, 6127–6138. 10.1021/acs.jctc.8b00391. 30354113

[ref50] Wan S.; Tresadern G.; Perez-Benito L.; Vlijmen H.; Coveney P. V. Accuracy and Precision of Alchemical Relative Free Energy Predictions with and without Replica-Exchange. Advanced Theory and Simulations 2020, 3, 1900195 10.1002/adts.201900195. PMC8427472 34527855

