# Supplementary material for: Ensembles Are Required to Handle Aleatoric and Parametric Uncertainty in Molecular Dynamics Simulation
Source: J Chem Theory Comput. 2021 Jul 19;17(8):5187–97. doi: 10.1021/acs.jctc.1c00526 (PMC8389531; doi:10.1021/acs.jctc.1c00526)
Supplement: Supplementary file 3 — ct1c00526_si_003.pdf [file ct1c00526_si_003.pdf]

Ensembles are required to handle aleatoric and parametric uncertainty in molecular dynamics simulation: Supplementary Information

Maxime Vassaux,[*,†] Shunzou Wan,[*,†] Wouter Edeling,[*,‡] and Peter V. Coveney[*,†,¶]

†*Centre for Computational Science, Department of Chemistry, University College London, London, United Kingdom*

‡*Centrum Wiskunde & Informatica, Scientific Computing Group, Amsterdam, The Netherlands*

¶*Informatics Institute, University of Amsterdam, Amsterdam, The Netherlands*

E-mail: m.vassaux@ucl.ac.uk; shunzhou.wan@ucl.ac.uk; wouter.edeling@cwi.nl; p.v.coveney@ucl.ac.uk

Introduction

The Supplementary Information contains results which provide further information on aspects of the uncertainty in the BAC workflow based on NAMD, along with details on the parameter refinement we performed.

Parameter distributions

Table S1 contains the 14 parameters which were included in the final UQ campaign. All were prescribed with uniform distributions with ranges displayed in Table S1, along with their default values, most of which can be found in NAMD documentation.[1]

Table S1: The parameters, with their default values and uncertain range, which were included in the final UQ campaign. The 'group' column indicates the group from which the parameter was selected, namely the physical parameters (P) or the solver parameters (S). A description of these parameters can be found in the NAMD software documentation.[1]

Parameter name	unit	default	min	max	group
Set Temperature (setTemperature)	K	300.00	280.00	320.00	P
Equilibration duration (time_factor_eq)	ns	1.86	1.58	2.14	P
Pressure (BerendsenPressureTarget)	bar	1.01325	0.86	1.17	P
Simulation duration (time_sim1)	ns	10.00	8.50	11.50	P
Minimum distance between protein and box edge (box_size)	A	14.00	11.90	16.10	S
Non-bonded interactions cut-off (cutoff)	A	12.00	10.20	13.80	S
Timestep duration (timestep)	fs	2.00	1.70	2.30	S
ShakeH bond-length error (rigidtolerance)	A	0.00001	0.0000085	0.000015	S
Particle Mesh Ewald grid spacing (PMEGridSpacing)	A	1.00	0.85	1.15	S
Initial temperature (initTemperature_eq1)	K	50.0	42.50	57.50	S
Temperature increment (reassingIncr_eq1)	K	1.00	0.85	1.15	S
Langevin Dynamics damping coefficient (langevinDamping)	ps^{-1}	5.00	4.25	5.75	S
Barostat compressibility (BerendsenPressureCompressibility)	bar^{-1}	0.000046	0.000039	0.000053	S
Barostat relaxation time (BerendsenPressureRelaxationTime)	fs	100.00	85.00	115.00	S

Parameter refinement

The dimension-adaptive method iteratively builds a sampling plan, using a linear combination of points from quadrature rules of different order, as the locations on which to evaluate BAC. All parameters are initialized with quadrature order zero and refinement is achieved by anisotropically increasing the quadrature order of (combinations of) parameters within a given iteration of the algorithm, based on a suitable error metric.

Consider Figure S1, which shows the colour-coded refinement per iteration. Specifically, each column shows the quadrature orders that were used to refine the sampling plan. The first column is fully white, as all parameters are initialised to a zero-order rule. In the second column one parameter is refined to first order, and from there on different parameters are refined. Note that S1 only shows the refinements related to the quadrature-order multi-indices $\mathbf{l} = (l_1, \cdots, l_d)$ in the accepted set Λ (see the Methods section of the main article). Once the refinement has completed, which in our case occurred when our computational budget of 2M core hours was depleted, we can also add all computed candidate $\mathbf{l}$ to Λ. This has the effect that we do not waste samples and that, in the final analysis step, every input is refined to at least first order. The Sobol indices described next are generated using this final sample set, since a first-order Sobol index of a parameter with a 0-th order rule will be zero.

Sensitivity analysis

Sensitivity analysis of the 14 parameters considered is performed computing Sobol indices (see figure S2), which translate the contribution of each parameter to the quantity of interest (QoI) variance. The sensitivity analysis is performed with an extended range of temperature that is 255K to 345K, resulting in a total of 123 parametric configurations simulated. First-order Sobol indices characterise the influence of individual parameters while high-order

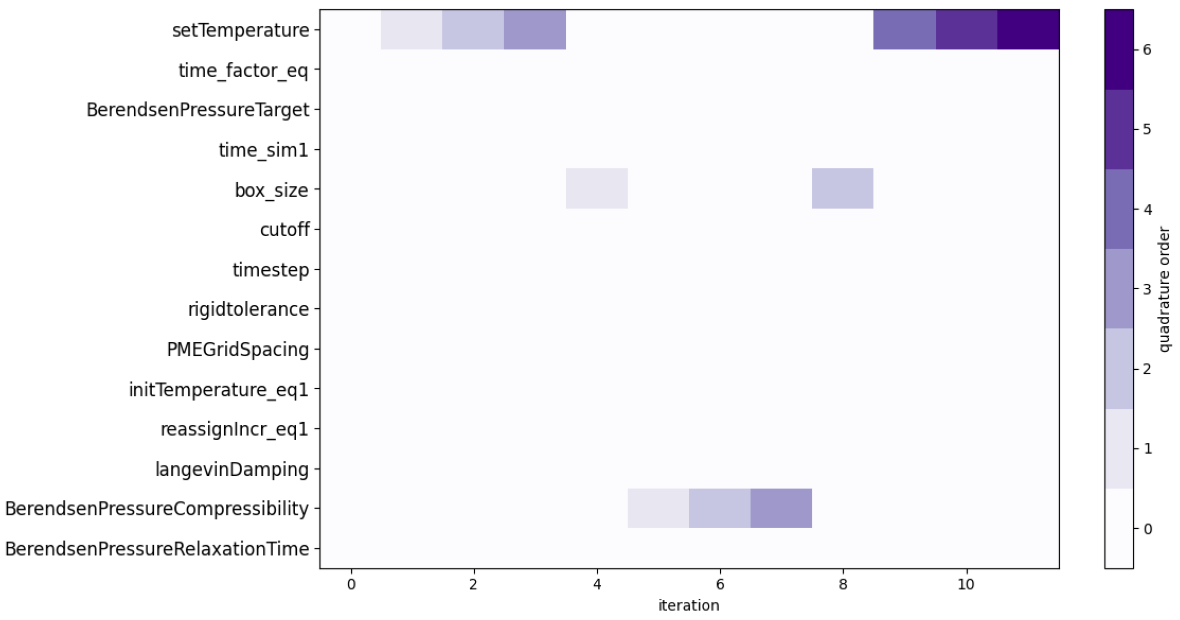

Figure S1: Colour-coded refinements per iteration of the dimension-adaptive algorithm.

indices characterise the coupled influence of multiple parameters. BAC predictions are mostly sensitive to the independent contributions of parameters. Indeed, the sum of first-order Sobol indices amounts to 0.997 (out of 1), that is they contribute to 99.7% of the QoI variance. Simultaneous variation of multiple parameters does not amplify uncertainty. In order, temperature (0.79), the volume (0.06), and the compressibility (0.05) of the simulated system are the most sensitive parameters. Temperature is the single most-influential parameter out of the 14 parameters analysed, contributing to almost 80% of the observed QoI variance. Uncertainty in the prescribed temperature of the system leads to variations of a few kcal/-mol of the computed binding energies. Consequently, predicted drug efficiency is extremely sensitive to the temperature at which binding occurs.

It is clear from the results of Figure S2 that the temperature is by far the most influential input parameter. In fact, it dominates to such an extent that it is hard to gauge the importance of the other parameters. For this reason, we computed an approximation to the first-order Sobol indices in case the temperature was not allowed to vary (assuming absolute

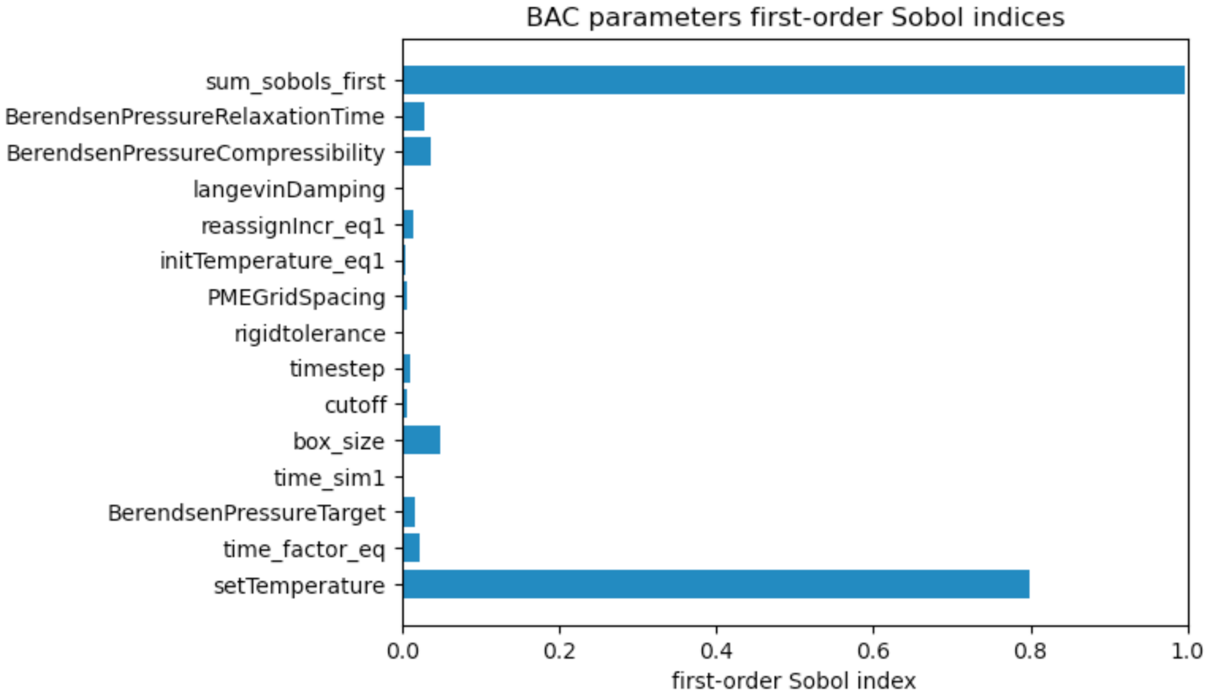

Figure S2: Variance-based sensitivity analysis of BAC. First-order Sobol indices of the 14 parameters considered in the sensitivity analysis. The sum of the first-order Sobol indices amounts to 0.997, leaving second-order Sobol indices negligible. In order, the temperature, the volume of the system and the compressibility are the three most influential parameters.

certainty in its value). In particular, we extracted an isotropic sparse grid[2] from the already simulated data, in which all parameters were refined once, except the temperature, which was kept fixed at 300K. This is a coarse approximation consisting of 27 points in parametric space, each one simulated with 25 replicas. More refined isotropic sparse grids are not a subset of the data we already have, and would require more expensive simulations. As such, the results only give a first-order approximation to the relevance of each parameter, when the temperature is not allowed to vary, see Figure S3. The box size is the most important as expected, along with the Berendsen barostat parameters.

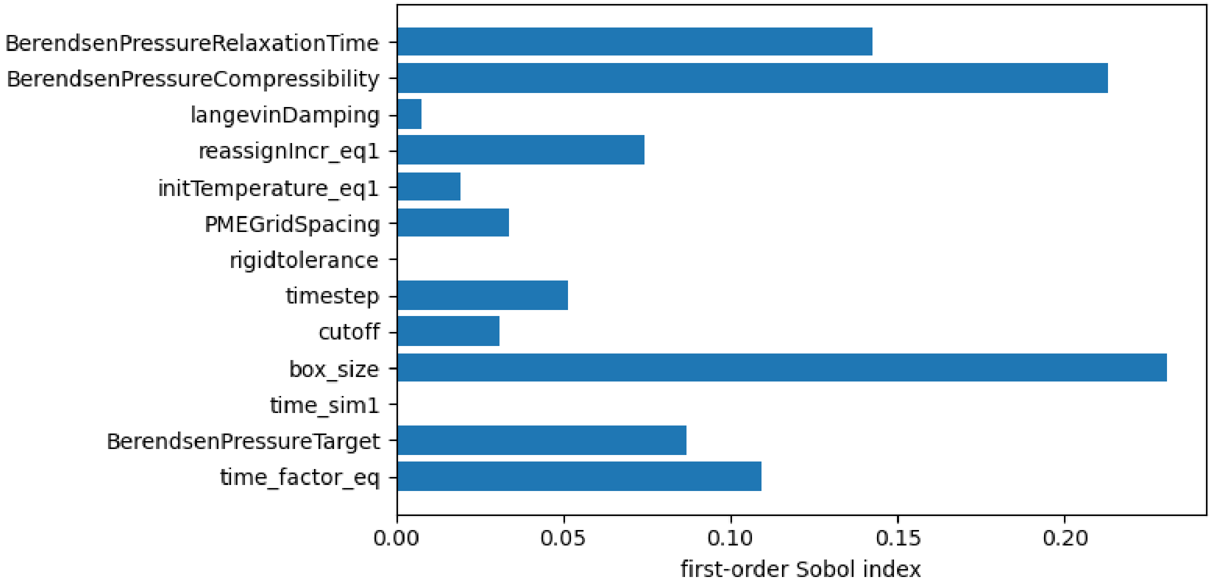

Figure S3: Variance-based sensitivity analysis of BAC at fixed 300K temperature. First-order Sobol indices of the 13 parameters remaining in the sensitivity analysis. The sum of all first-order Sobol indices is not included. A sparse grid in which each input is refined once while keeping all other parameters fixed is used, therefore higher-order interaction effects between parameters are not estimated and the first-order Sobol indices sum to one.

Uncertainty quantification

The UQ campaign results for the extended range of temperatures introduced in the previous section are shown in the figure S4. The consideration of a larger range of variation of temperature ($[255, 345]$) leads to the observation of a second mode in the binding energy distribution. This shows that a larger uncertainty in temperature could further induce unrealistic conformations, and persistent erroneous estimation of the binding free energy.

Complete parameter listing

The BAC workflow is a succession of four simulation phases: 3 equilibration phases (*eq0*, *eq1* and *eq2*) and one simulation phase (*sim1*). The complete listing of the 175 parameters found in the four phases of the BAC workflow as well as their type and default values are found in the following JSON list:

- For phase *eq0*:

```
{
  "paraTypeCharmm_eq0": {
    "default": "off",
    "type": "string"
  },
  "amber_eq0": {
    "default": "yes",
    "type": "string"
  },
  "parmfile_eq0": {
    "default": "../build/complex.prmtop",
```

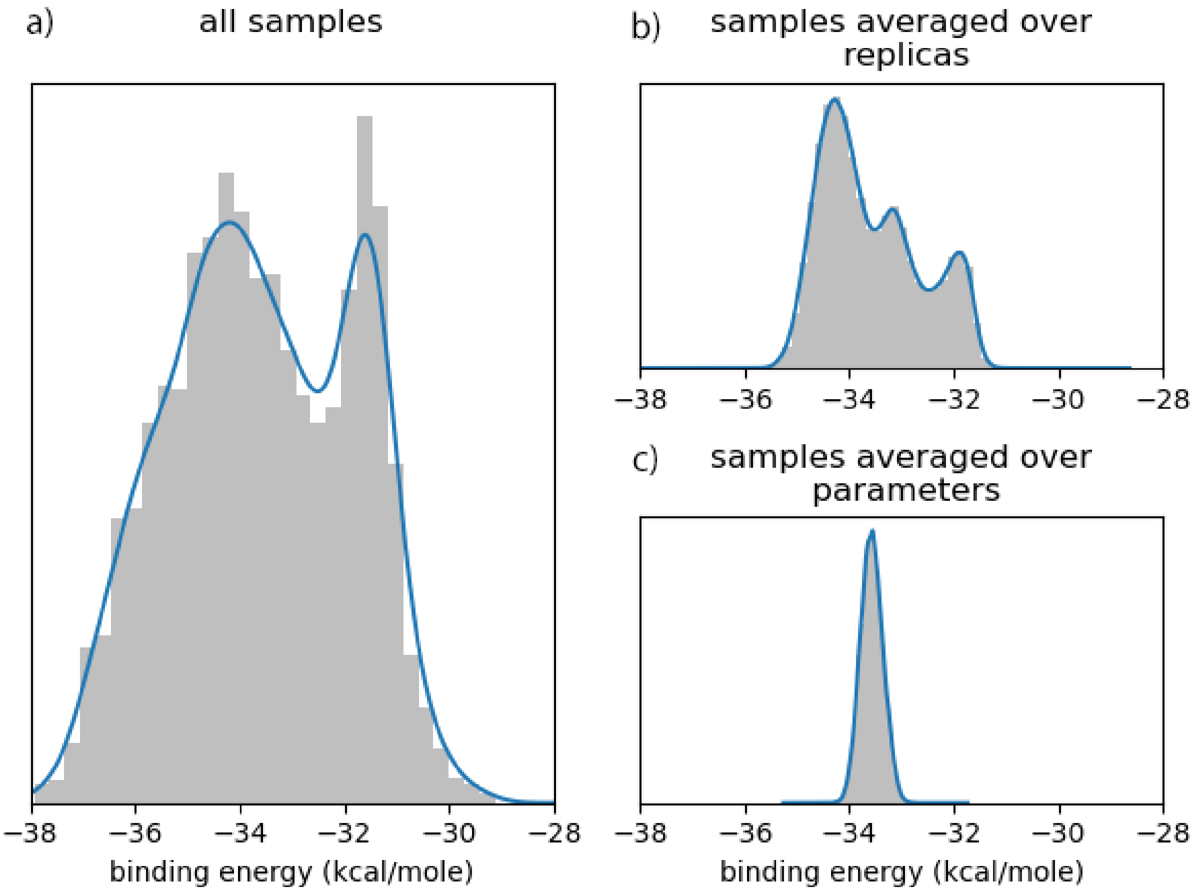

Figure S4: **Distribution of computed binding free energies.** (a) Distribution of the binding energy computed for each replica of each parametric configuration, resulting in 1575 samples in total. (b) Distribution of the binding energies averaged over the 123 parametric configurations for each of the 25 replicas. The distribution shows the influence of aleatoric uncertainty on the computed binding energies. (c) Distribution of the binding energies averaged over the 25 replicas for each of the 123 parametric configurations. The distribution shows the influence of parametric uncertainty on the computed binding energies.

```
    "type": "string"
  },
  "coordinates_eq0": {
    "default": "../build/complex.pdb",
    "type": "string"
  },
  "readexclusions_eq0": {
    "default": "no",
    "type": "string"
  },
  "exclude_eq0": {
    "default": "scaled1-4",
    "type": "string"
  },
  "_1_4scaling_eq0": {
    "default": 0.833333,
    "type": "float"
  },
  "cutoff_eq0": {
    "default": 12,
    "type": "float"
  },
  "switching_eq0": {
    "default": "on",
    "type": "string"
  },
```

```
  "switchdist_eq0": {
    "default": 10,
    "type": "float"
  },
  "pairlistdist_eq0": {
    "default": 13.5,
    "type": "float"
  },
  "timestep_eq0": {
    "default": 2,
    "type": "float"
  },
  "rigidBonds_eq0": {
    "default": "all",
    "type": "string"
  },
  "rigidtolerance_eq0": {
    "default": 0.00001,
    "type": "float"
  },
  "rigidIterations_eq0": {
    "default": 100,
    "type": "float"
  },
  "nonbondedFreq_eq0": {
    "default": 1,
```

```
    "type": "float"
  },
  "fullElectFrequency_eq0": {
    "default": 2,
    "type": "float"
  },
  "stepspercycle_eq0": {
    "default": 10,
    "type": "float"
  },
  "wrapWater_eq0": {
    "default": "on",
    "type": "string"
  },
  "wrapAll_eq0": {
    "default": "on",
    "type": "string"
  },
  "outputEnergies_eq0": {
    "default": 500,
    "type": "float"
  },
  "outputPressure_eq0": {
    "default": 500,
    "type": "float"
  },
```

```json
"source_eq0": {
  "default": "./dim0.conf",
  "type": "string"
},
"PME_eq0": {
  "default": "yes",
  "type": "string"
},
"PMEGridSpacing_eq0": {
  "default": 1,
  "type": "float"
},
"binaryoutput_eq0": {
  "default": "yes",
  "type": "string"
},
"constraints_eq0": {
  "default": "on",
  "type": "string"
},
"consexp_eq0": {
  "default": 2,
  "type": "float"
},
"consref_eq0": {
  "default": "../build/complex.pdb",
```

```
    "type": "string"
  },
  "conskfile_eq0": {
    "default": "../constraint/cons.pdb",
    "type": "string"
  },
  "conskcol_eq0": {
    "default": "O",
    "type": "string"
  },
  "temperature_eq0": {
    "default": 50,
    "type": "float"
  },
  "set_factor_eq0": {
    "default": 10,
    "type": "float"
  },
  "set_nall_eq0": {
    "default": 0,
    "type": "float"
  },
  "set_n_eq0": {
    "default": 1,
    "type": "float"
  },
```

```
  "minimization_eq0": {
    "default": "on",
    "type": "string"
  },
  "constraintScaling_eq0": {
    "default": 0,
    "type": "float"
  },
  "minimize_eq0": {
    "default": 1000,
    "type": "float"
  }
```

- For phase *eq1*:

```
{

  "paraTypeCharmm_eq1": {
    "default": "off",
    "type": "string"
  },
  "amber_eq1": {
    "default": "yes",
    "type": "string"
  },
  "parmfile_eq1": {
    "default": "../build/complex.prmtop",
```

```
    "type": "string"
  },
  "readexclusions_eq1": {
    "default": "no",
    "type": "string"
  },
  "coordinates_eq1": {
    "default": "../build/complex.pdb",
    "type": "string"
  },
  "exclude_eq1": {
    "default": "scaled1-4",
    "type": "string"
  },
  "_1_4scaling_eq1": {
    "default": 0.833333,
    "type": "float"
  },
  "cutoff_eq1": {
    "default": 12,
    "type": "float"
  },
  "switching_eq1": {
    "default": "on",
    "type": "string"
  },
```

```
  "switchdist_eq1": {
    "default": 10,
    "type": "float"
  },
  "pairlistdist_eq1": {
    "default": 13.5,
    "type": "float"
  },
  "timestep_eq1": {
2

    "default": 2,
    "type": "float"
  },
  "rigidBonds_eq1": {
    "default": "all",
    "type": "string"
  },
  "rigidtolerance_eq1": {
    "default": 0.00001,
    "type": "float"
  },
  "rigidIterations_eq1": {
    "default": 100,
    "type": "float"
  },
```

```
"nonbondedFreq_eq1": {
  "default": 1,
  "type": "float"
},
"fullElectFrequency_eq1": {
  "default": 2,
  "type": "float"
},
"stepspercycle_eq1": {
  "default": 10,
  "type": "float"
},
"wrapWater_eq1": {
  "default": "on",
  "type": "string"
},
"wrapAll_eq1": {
  "default": "on",
  "type": "string"
},
"outputEnergies_eq1": {
  "default": 500,
  "type": "float"
},
"outputPressure_eq1": {
  "default": 500,
```

```
    "type": "float"
  },
  "PME_eq1": {
    "default": "yes",
    "type": "string"
  },
  "PMEGridSpacing_eq1": {
    "default": 1,
    "type": "float"
  },
  "constraints_eq1": {
    "default": "on",
    "type": "string"
  },
  "consexp_eq1": {
    "default": 2,
    "type": "float"
  },
  "consref_eq1": {
    "default": "../build/complex.pdb",
    "type": "string"
  },
  "conskfile_eq1": {
    "default": "../constraint/cons.pdb",
    "type": "string"
  },
```

```
"conskcol_eq1": {
  "default": "0",
  "type": "string"
},
"constraintScaling_eq1": {
  "default": 1,
  "type": "float"
},
"temperature_eq1": {
  "default": 50,
  "type": "float"
},
"reassignFreq_eq1": {
  "default": 100,
  "type": "float"
},
"reassignIncr_eq1": {
  "default": 1,
  "type": "float"
},
"reassignHold_eq1": {
  "default": 300,
  "type": "float"
},
"binaryoutput_eq1": {
  "default": "yes",
```

```
    "type": "string"
  },
  "binaryrestart_eq1": {
    "default": "yes",
    "type": "string"
  },
  "restartfreq_eq1": {
    "default": 500000,
    "type": "float"
  },
  "xstFreq_eq1": {
    "default": 5000,
    "type": "float"
  },
  "dcdfreq_eq1": {
    "default": 5000,
    "type": "float"
  },
  "DCDUnitcell_eq1": {
    "default": "yes",
    "type": "string"
  },
  "run_eq1": {
    "default": 30000,
    "type": "float"
  }
```

- For phase *eq2*:

```
{

  "paraTypeCharmm_eq2": {
    "default": "off",
    "type": "string"
  },
  "amber_eq2": {
    "default": "yes",
    "type": "string"
  },
  "parmfile_eq2": {
    "default": "../build/complex.prmtop",
    "type": "string"
  },
  "readexclusions_eq2": {
    "default": "no",
    "type": "string"
  },
  "coordinates_eq2": {
    "default": "../build/complex.pdb",
    "type": "string"
  },
  "exclude_eq2": {
```

```
    "default": "scaled1-4",
    "type": "string"
  },
  "_1_4scaling_eq2": {
    "default": 0.833333,
    "type": "float"
  },
  "cutoff_eq2": {
    "default": 12,
    "type": "float"
  },
  "switching_eq2": {
    "default": "on",
    "type": "string"
  },
  "switchdist_eq2": {
    "default": 10,
    "type": "float"
  },
  "pairlistdist_eq2": {
    "default": 13.5,
    "type": "float"
  },
  "timestep_eq2": {
    "default": 2,
    "type": "float"
```

```
  },
  "rigidBonds_eq2": {
    "default": "all",
    "type": "string"
  },
  "rigidtolerance_eq2": {
    "default": 0.00001,
    "type": "float"
  },
  "rigidIterations_eq2": {
    "default": 100,
    "type": "float"
  },
  "nonbondedFreq_eq2": {
    "default": 1,
    "type": "float"
  },
  "fullElectFrequency_eq2": {
    "default": 2,
    "type": "float"
  },
  "stepspercycle_eq2": {
    "default": 10,
    "type": "float"
  },
  "wrapWater_eq2": {
```

```json
    "default": "on",
    "type": "string"
  },
  "wrapAll_eq2": {
    "default": "on",
    "type": "string"
  },
  "outputEnergies_eq2": {
    "default": 500,
    "type": "float"
  },
  "outputPressure_eq2": {
    "default": 500,
    "type": "float"
  },
  "PME_eq2": {
    "default": "yes",
    "type": "string"
  },
  "PMEGridSpacing_eq2": {
    "default": 1,
    "type": "float"
  },
  "constraints_eq2": {
    "default": "on",
    "type": "string"
```

```
},
"consexp_eq2": {
  "default": 2,
  "type": "float"
},
"consref_eq2": {
  "default": "../build/complex.pdb",
  "type": "string"
},
"conskfile_eq2": {
  "default": "../constraint/cons.pdb",
  "type": "string"
},
"conskcol_eq2": {
  "default": "O",
  "type": "string"
},
"langevin_eq2": {
  "default": "on",
  "type": "string"
},
"langevinDamping_eq2": {
  "default": 5,
  "type": "float"
},
"langevinTemp_eq2": {
```

```
    "default": 300,
    "type": "float"
  },
  "langevinHydrogen_eq2": {
    "default": "no",
    "type": "string"
  },
  "useGroupPressure_eq2": {
    "default": "yes",
    "type": "string"
  },
  "useFlexibleCell_eq2": {
    "default": "no",
    "type": "string"
  },
  "useConstantArea_eq2": {
    "default": "no",
    "type": "string"
  },
  "BerendsenPressure_eq2": {
    "default": "on",
    "type": "string"
  },
  "BerendsenPressureTarget_eq2": {
    "default": 1,
    "type": "float"
```

```
  },
  "BerendsenPressureCompressibility_eq2": {
    "default": 0.0000457,
    "type": "float"
  },
  "BerendsenPressureRelaxationTime_eq2": {
    "default": 100,
    "type": "float"
  },
  "BerendsenPressureFreq_eq2": {
    "default": 2,
    "type": "float"
  },
  "binaryoutput_eq2": {
    "default": "yes",
    "type": "string"
  },
  "binaryrestart_eq2": {
    "default": "yes",
    "type": "string"
  },
  "restartfreq_eq2": {
    "default": 500000,
    "type": "float"
  },
  "xstFreq_eq2": {
```

```
    "default": 5000,
    "type": "float"
  },
  "dcdfreq_eq2": {
    "default": 5000,
    "type": "float"
  },
  "DCDUnitcell_eq2": {
    "default": "yes",
    "type": "string"
  },
  "set_factor_eq2": {
    "default": 1,
    "type": "float"
  },
  "set_nall_eq2": {
    "default": 0,
    "type": "float"
  },
  "set_n_eq2": {
    "default": 1,
    "type": "float"
  },
  "set_run2_eq2": {
    "default": 40000,
    "type": "float"
```

```
  },
  "constraintScaling_eq2": {
    "default": 0,
    "type": "float"
  },
  "run_eq2": {
    "default": 60000,
    "type": "float"
  }
```

- For phase *sim1*:

```
{

  "paraTypeCharmm_sim1": {
    "default": "off",
    "type": "string"
  },
  "amber_sim1": {
    "default": "yes",
    "type": "string"
  },
  "parmfile_sim1": {
    "default": "../build/complex.prmtop",
    "type": "string"
  },
  "readexclusions_sim1": {
```

```
    "default": "no",
    "type": "string"
  },
  "coordinates_sim1": {
    "default": "../build/complex.pdb",
    "type": "string"
  },
  "exclude_sim1": {
    "default": "scaled1-4",
    "type": "string"
  },
  "_1_4scaling_sim1": {
    "default": 0.833333,
    "type": "float"
  },
  "cutoff_sim1": {
    "default": 12,
    "type": "float"
  },
  "switching_sim1": {
    "default": "on",
    "type": "string"
  },
  "switchdist_sim1": {
    "default": 10,
    "type": "float"
```

```
},
"pairlistdist_sim1": {
  "default": 13.5,
  "type": "float"
},
"timestep_sim1": {
  "default": 2,
  "type": "float"
},
"rigidBonds_sim1": {
  "default": "all",
  "type": "string"
},
"rigidtolerance_sim1": {
  "default": 0.00001,
  "type": "float"
},
"rigidIterations_sim1": {
  "default": 100,
  "type": "float"
},
"nonbondedFreq_sim1": {
  "default": 1,
  "type": "float"
},
"fullElectFrequency_sim1": {
```

```
    "default": 2,
    "type": "float"
  },
  "stepspercycle_sim1": {
    "default": 10,
    "type": "float"
  },
  "wrapWater_sim1": {
    "default": "on",
    "type": "string"
  },
  "wrapAll_sim1": {
    "default": "on",
    "type": "string"
  },
  "outputEnergies_sim1": {
    "default": 500,
    "type": "float"
  },
  "outputPressure_sim1": {
    "default": 5000,
    "type": "float"
  },
  "PME_sim1": {
    "default": "yes",
    "type": "string"
```

```
},
"PMEGridSpacing_sim1": {
  "default": 1,
  "type": "float"
},
"binaryoutput_sim1": {
  "default": "yes",
  "type": "string"
},
"binaryrestart_sim1": {
  "default": "yes",
  "type": "string"
},
"restartfreq_sim1": {
  "default": 500000,
  "type": "float"
},
"xstFreq_sim1": {
  "default": 5000,
  "type": "float"
},
"langevin_sim1": {
  "default": "on",
  "type": "string"
},
"langevinDamping_sim1": {
```

```
    "default": 5,
    "type": "float"
  },
  "langevinTemp_sim1": {
    "default": 300,
    "type": "float"
  },
  "langevinHydrogen_sim1": {
    "default": "no",
    "type": "string"
  },
  "useGroupPressure_sim1": {
    "default": "yes",
    "type": "string"
  },
  "useFlexibleCell_sim1": {
    "default": "no",
    "type": "string"
  },
  "useConstantArea_sim1": {
    "default": "no",
    "type": "string"
  },
  "BerendsenPressure_sim1": {
    "default": "on",
    "type": "string"
```

```
  },
  "BerendsenPressureTarget_sim1": {
    "default": 1.01325,
    "type": "float"
  },
  "BerendsenPressureCompressibility_sim1": {
    "default": 0.0000457,
    "type": "float"
  },
  "BerendsenPressureRelaxationTime_sim1": {
    "default": 100,
    "type": "float"
  },
  "BerendsenPressureFreq_sim1": {
    "default": 2,
    "type": "float"
  },
  "dcdfreq_sim1": {
    "default": 5000,
    "type": "float"
  },
  "DCDUnitcell_sim1": {
    "default": "yes",
    "type": "string"
  },
  "run_sim1": {
```

```
    "default": 50000,
    "type": "float"
  }
```

References

(1) NAMD 2.14 User's Guide. https://www.ks.uiuc.edu/Research/namd/2.14/ug/.

(2) , S.A. Smolyak, Quadrature and interpolation formulas for tensor products of certain classes of functions, Doklady Akademii Nauk, 148, 5, 1042–1045, 1963.
